# RETRACTED: Ibrahim et al. Hepatoprotective Effects of Biochanin A on Thioacetamide-Induced Liver Cirrhosis in Experimental Rats. *Molecules* 2023, *28*, 7608

**DOI:** 10.3390/molecules30244801

**Published:** 2025-12-17

**Authors:** Mohamed Yousif Ibrahim, Zaenah Zuhair Alamri, Ameena S. M. Juma, Sarah Ashour Hamood, Suhayla Hamad Shareef, Mahmood Ameen Abdulla, Soher Nagi Jayash

**Affiliations:** 1 Faculty of Pharmacy, Elrazi University, Khartoum 11115, Sudan; 2 Department of Biological Sciences, Faculty of Science, University of Jeddah, Jeddah 21589, Saudi Arabia; zzalamri@uj.edu.sa; 3 Department of Medical Microbiology, College of Science, Cihan University-Erbil, Erbil 44001, Iraq; ameena.sabah@cihanuniversity.edu.iq (A.S.M.J.); mahmood.ameen@cihanuniversity.edu.iq (M.A.A.); 4 Biomedical Engineering Department, Al-Essra University College, Baghdad 10011, Iraq; sara.ashour@esraa.edu.iq; 5 Department of Biology, College of Education, Salahaddin University-Erbil, Erbil 44001, Iraq; suhayla.shareef@su.edu.krd; 6 The Roslin Institute and Royal (Dick) School of Veterinary Studies, University of Edinburgh, Easter Bush Campus, Midlothian EH25 9RG, UK

The Journal retracts the article “Hepatoprotective Effects of Biochanin A on Thioacetamide-Induced Liver Cirrhosis in Experimental Rats” [1].

Following publication, concerns were brought to the attention of the Editorial Office regarding the integrity of an image presented in this article [1].

Adhering to our standard procedure, an investigation was conducted by the Editorial Office and Editorial Board that confirmed indications of inappropriate editing and duplication between the sub-images in Figure 5 [1] and the sub-figures in Figure 4 of previously published articles [2,3], representing different experimental conditions. This investigation considered a report from the University of Edinburgh, which further validated the above-mentioned concerns and indicated that Dr. Soher Nagi Jayash was not aware of the image concerns at any stage of the submission or publication process. Consequently, the Editorial Board has lost confidence in the reliability of the findings and has decided to retract this publication [1], as per MDPI’s retraction policy (https://www.mdpi.com/ethics#_bookmark30).

This retraction was approved by the Editor-in-Chief of the journal *Molecules.*

Soher Nagi Jayash and Suhayla Hamad Shareef agreed to this retraction. The remaining authors did not provide a comment on this decision.
